# A Novel Treatment Approach of Ultrasound-Guided Radiofrequency Ablation of the Greater Trochanteric Sensory Nerve for Recalcitrant Greater Trochanteric Pain Syndrome

**DOI:** 10.7759/cureus.19859

**Published:** 2021-11-24

**Authors:** Yin-Ting Chen, Christine M Olanrewaju

**Affiliations:** 1 Department of Rehabilitation, Uniformed Services University of the Health Sciences, Bethesda, USA; 2 Department of Orthopaedics and Rehabilitation, Walter Reed National Military Medical Center, Bethesda, USA; 3 Physical Medicine and Rehabilitation, Landstuhl Regional Medical Center, APO, USA

**Keywords:** chronic pain, radiofrequency ablation, greater trochanteric sensory nerve, greater trochanteric pain syndrome, hip pain

## Abstract

This report describes a novel technique for the treatment of recalcitrant greater trochanteric pain syndrome (GPTS) by radiofrequency ablation (RFA) of the greater trochanteric sensory nerve (GTsn). Here, we describe one patient suffering from recalcitrant GTPS treated with RFA of the GTsn in the outpatient pain clinic setting. Over the eight months subsequent to treatment, the patient was monitored for changes in the Numerical Pain Rating (NPR) and Lower Extremity Functional Score (LEFS). The patient demonstrated meaningful symptomatic and functional improvement as measured by both NPR and LEFS. GTsn RFA may be a viable treatment option for recalcitrant GTPS. Larger comparative trials are needed to establish improved results over conventional treatments.

## Introduction

Greater trochanteric pain syndrome (GTPS) is a common cause of lateral hip pain. GTPS is characterized by the presence of pain in the greater trochanter (GT) region. It can negatively impact ambulation, activities, and sleep [1]. GTPS is more frequently found in females in the fifth decade of life or young athletic males without intra-articular pathology of the hip [2,3]. Although GT bursitis has long been thought to be the cause of GTPS, gluteus minimus, medius tendinopathy, or tendon tear are the most common causes [4-6]. The prevalence of GTPS is estimated to be 10-25% of the general population in developed nations [7,8].

GTPS generally responds well to conservative treatment. First-line treatments for GTPS include activity modification, nonsteroidal anti-inflammatory drugs (NSAIDs), and physiotherapy [9]. Corticosteroid injection is a common treatment; however, there are conflicting reports as to its efficacy. Barratt et al. found a lack of evidence to support the use of fluoroscopically guided GT bursa steroid injection in their systematic review, whereas Torres et al. found this technique to be effective and recommended it as a second-line treatment [9,10]. Other common interventions include shockwave therapy and platelet-rich plasma (PRP) injections, although there is some conflicting evidence to the contrary [10-12]. An estimated 10% of patients with GTPS remain recalcitrant to conservative treatment, after which surgical treatment may be the next successive option for these patients [13,14]. Surgical treatments include GT bursectomy, gluteal tendon tear repair, iliotibial band (ITB) lengthening/release, or trochanteric reduction osteotomy, all of which are invasive and may require long periods of postoperative recovery and rehabilitation [15].

Radiofrequency ablation (RFA) is a common treatment modality for chronic musculoskeletal pain conditions. RFA is conducted by placing a radiofrequency (RF) probe in direct contact with the target terminal sensory nerve to the painful structure with subsequent induction of thermocoagulation, thereby dampening or severing the sensory connection from the painful structure. RFA treatment is well established for the treatment of knee pain, facetogenic back pain, and hip pain [16,17]. However, secondary to a lack of specific terminal sensory nerves as targets, there are scant reports of RFA treatment for painful extra-articular structures such as bursa, tendons, or periosteum. To our knowledge, to date, there are no published descriptions of ultrasound-guided RFA treatment for GTPS.

The terminal sensory innervation of GT structures was relatively uncertain until 2012 when Genth et al. published their study identifying a small terminal sensory nerve branch innervating GT structures in seven adult cadavers [18]. This GT sensory nerve (GTsn) was described as a femoral nerve branch, entering the posterior gluteal space along with the medial femoral circumflex artery (MFCA) and coursing inferiorly to the tendon of the inferior gemellus (IG) muscle in the ischiofemoral interval to reach the GT periosteum and bursal structures [18]. Histological examination of a formalin-fixed fetal cadaver showed that the GTsn mainly comprises small, unmyelinated nerve fibers with lesser amounts of thinly myelinated fibers, consistent with sensory nerve fibers [18]. Notably, the authors did not identify any other nerves entering the GT structures from nearby nerves such as the superior gluteal nerve, inferior gluteal nerve, or the sciatic nerve, suggesting that the GTsn is likely the sole source of sensory innervation to the GT structures. The authors postulated that their finding would potentially open new therapeutic approaches to treat recalcitrant GTPS, but, to date, there are no reports of GTPS treatment via ultrasound-guided nerve block or ablation, neuromodulation, or surgical neurectomy of the GTsn [18]. In this report, we describe the technique we have developed to identify the GTsn under ultrasound guidance and to perform ultrasound-guided GTsn RFA for the treatment of recalcitrant GTPS.

## Technical report

Ultrasound identification of greater trochanteric sensory nerve

**Figure 1:**
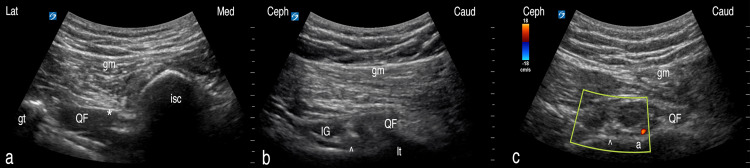
The scanning and identification of the GTsn. (a) Transverse view of the ischiofemoral interval, the sciatic nerve is visualized just superficial to the quadratus femoris. (b) Transverse axis view to the quadratus femoris muscle obtained by placing the transducer long-axis to the femur; the transducer is then moved laterally to the sciatic nerve (no longer in view). The GTsn is in view in the fascial plane between the inferior gemellus and the quadratus femoris. (c) Color-flow Doppler demonstrates the presence of the MFCA. *: lesser trochanter (lt), quadratus femoris (QF), sciatic nerve; ^: ischial tuberosity (isc), inferior gemellus (IG), GTsn; a: MFCA GTsn: greater trochanteric sensory nerve; MFCA: medial femoral circumflex artery

To identify the GTsn using ultrasound, the patient should be first placed in a prone position with hips in maximal internal rotation. A curvilinear transducer is then placed in the inferior posterior gluteal region in the transverse axis to the ischium at the level of the ischial tuberosity to visualize the quadratus femoris muscle (QF) and the sciatic nerve (Figure 1a). The transducer is then turned long-axis to the ischium to demonstrate the QF and the IG muscles in the transverse axis within the ischiofemoral interval. Then, the transducer is moved by a short-axis slide laterally until it is positioned just medial to the lesser trochanter (Figure 1b). In this view, (1) the IG appears as a medium-sized round terminal tendon with appropriate tendinous echotexture, and (2) the QF appears as rectangular hypoechoic muscle with minimal striation, separated by a dim fascial plane. The GTsn and the MFCA can be observed in the deep portion of this fascial plane on the posterior surface of the femoral neck and in close proximity to each other. The GTsn appears as a small-to-medium caliber nerve containing multiple fascicles with dimly hyperechoic epineurium. The MFCA is appreciated as a nearby small-caliber artery, best demonstrated using color-flow Doppler (Figure 1c). The lesser trochanter may be viewed just caudal to the MFCA and the GTsn if the hip is placed in an externally rotated position.

Illustrative case

This case describes a 30-year-old female with a two-year history of persistent pain in the GT region. The patient was experiencing significant functional limitations in that she was unable to perform the physical requirements of her occupation as a soldier in the US Army, including running, hiking terrain, and marching in formation. The patient was otherwise healthy, with a medical history notable only for hip pain. Social history was negative for alcohol, tobacco, or recreational drug use. Her hobbies included running, yoga, and pilates. Family history was unknown. The patient’s physical examination was notable for a height of 63 inches (160 cm) and a weight of 110 pounds (49.9 kg), with tenderness to palpation at the greater trochanter and just posteriorly; special tests included positive C-sign, negative Stinchfield, negative flexion, abduction, and external rotation (FABER)/flexion, adduction, and internal rotation (FADIR), and negative piriformis tests. There was an absence of motor weakness, atrophy, or other neurological signs. Both magnetic resonance imaging and ultrasound examinations were notable for chronic gluteal tendinopathy. Her pain did not improve with standard conservative treatments including physical therapy, GT bursa corticosteroid injection, and PRP treatment.

**Figure 2:**
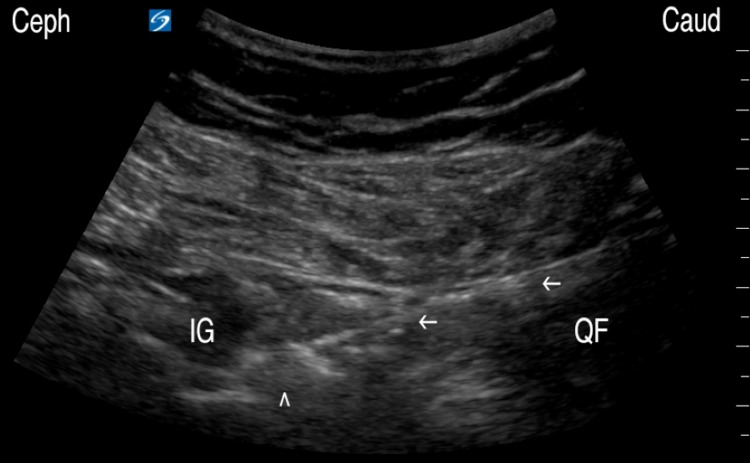
Needle or RF probe approach to the GTsn. IG: inferior gemellus; QF: quadratus femoris; RF: radiofrequency; GTsn: greater trochanteric sensory nerve ^: inferior gemellus, GTsn, ←: lesser trochanter, needle/probe

A diagnostic block was first performed in the following manner. The posterior gluteal region was prepared with chlorhexidine solution and the procedure was conducted under sterile techniques. An 8-3 MHz curvilinear transducer (C35xp transducer, Sonosite X-Porte, Fujifilm, Bothell, WA) was covered with a sterile probe cover and used to visualize anatomic structures during the procedure. Local anesthesia was achieved by injecting 1% lidocaine about the inferior gluteal fold area using a 27-gauge, 1-1/2-inch needle. After the GTsn was identified using the ultrasound technique described above, a 22-gauge, 3-1/2-inch needle was guided in an in-plane, caudal-to-cephalad approach until reaching the GTsn, followed by injecting 0.5 mL of 2% lidocaine and 0.5 mL of 0.75% bupivacaine (Figure 2), leading to an immediate pain reduction by 50%.

She returned one week later for the RFA treatment. Using the same technique as above described, a 20-gauge, 150 mm RF probe (RFP-100A RF Puncture Generator, Baylis Medical, Montreal, Canada) was placed in direct contact with the GTsn. The active tip of the RF probe was carefully positioned to maintain direct contact with GTsn while maximizing the distance to the MFCA. Sensory testing was positive at <0.5 V at 50 Hz and motor testing was negative at 1.5 V at 2 Hz. Subsequently, a mixture of 0.5 mL of 2% lidocaine and 0.5 mL of 0.75% bupivacaine was injected and allowed 90 seconds to establish pre-ablation analgesia. With continuous ultrasound visualization of the RF probe in the proper position, RFA was performed at one cycle of 85°C for 90 seconds, followed by the injection of 0.5 mL of 2% lidocaine, 0.5 mL of 0.75% bupivacaine, and 1.0 mL (10 mg/mL) of dexamethasone. Patient follow-up was done at four weeks, eight weeks, twelve weeks, four months, and eight months. The Numerical Pain Rating (NPR) and Lower Extremity Functional Score (LEFS) were obtained at baseline and each of the follow-up visits. The patient did not experience any complications during or subsequent to the procedure and noted steady improvement in pain and function in the following weeks. By four weeks post-procedure, the NPR improved from 6/10 to 0-3/10, and the LEFS improved from baseline 48 to 60 (out of 80). By eight weeks, she was able to again run 4 miles at a rigorous pace, front squat 22.7 kg (50 lbs), and the LEFS score further increased to 70 (out of 80). She was able to return to her profession as a US Army soldier without physical activity restrictions. At eight months post-procedure, she continued to have only 0-3/10 NPR during intense exertion without any functional limitations, with the LEFS score of 72 out of 80 (Table 1).

**Table 1:** The NPR and LEFS at baseline and follow-up visits. NPR: Numeric Pain Rating; LEFS: Lower Extremity Functional Score

	Baseline	Four weeks	Eight weeks	Twelve weeks	Four months	Eight months
NPR (out of 10)	6	0–3	0–3	0–3	1–2	0–3
LEFS (out of 80)	48	60	70	70	72	70

## Discussion

There is one prior report of potential GT sensory innervation. Dunn et al. described a sensory nerve branch of the inferior gluteal nerve to the superficial and deep GT bursae in two out of 16 cadaveric specimens [19]. Genth et al. were unable to replicate this finding in their study, nor did they find any other sensory branches arising from other nearby nerves [18]. Albeit the sample size was small, Genth et al. consistently identified the GTsn in all of their specimens, leading them to postulate that the sensory branch from the inferior gluteal nerve was likely an anatomic variant [18]. In our review of the literature, we did not find any reports of treatments to the proposed sensory branch of the inferior gluteal nerve based on the Dunn et al. study.

The gluteus minimus and medius tendons are the primary pain generator in GTPS. Periarticular tendons may receive sensory innervation from joint articular sensory nerves. Eckmann et al. reported successful treatment of chronic rotator cuff tendinopathy by RFA of the glenohumeral joint articular nerves; however, there are no analogous reports of hip articular nerve RFA being therapeutic for GTPS [20]. Tendons are also known to receive sensory innervation from the nearby motor, cutaneous, and peritendinous nerves, and few dedicated terminal sensory nerves to osteotendinous structures have been reported [21]. For example, successful treatment of recalcitrant medial and lateral epicondylopathy by surgical neurectomy of the dedicated terminal sensory nerves to both the lateral and medial epicondyles have been reported [22,23]. Although Genth et al. did not specifically demonstrate these tendons being innervated by the GTsn, given that the GTsn infiltrated the GT periosteum and GT bursa, it is reasonable that it may also innervate the gluteal tendons given its peritendinous targets, as reflected by the response in our case. Surgical denervation of GTsn may not be ideal due to its depth and the complex neurovascular anatomy of the deep gluteal space. Our technique allows highly accurate and selective denervation of the GTsn through RFA.

The MFCA is a major vascular supply for the femoral head and femoral neck. Although there are detailed anatomic studies of the MFCA, there are no prior descriptions of the accompanying GTsn [24,25]. Our ultrasound examination showed that the GTsn is a small-to-medium caliber nerve with multiple fascicles and travels along with the MFCA in the fascial plane between the IG and QF. In our experience, we found the distance between the MFCA and GTsn to be approximately 5-8 mm. The GTsn is readily visualized by ultrasound despite its depth. As the MFCA has a predictable course, other image guidance modalities such as computed tomography or fluoroscopy may be feasible if specific bony landmarks or image measurements are characterized and validated. However, without direct visualization, the risk of injuring the MFCA during RFA is a concern. As the GTsn is a deep structure, it may be difficult to visualize by ultrasound due to patient body habits or the limitation of the ultrasound machine. Inability to adequately visualize the GTsn and/or the MFCA and close proximity between the GTsn and the MFCA are contraindications to neuroablative treatments such as RFA or chemoneurolysis.

**Figure 3:**
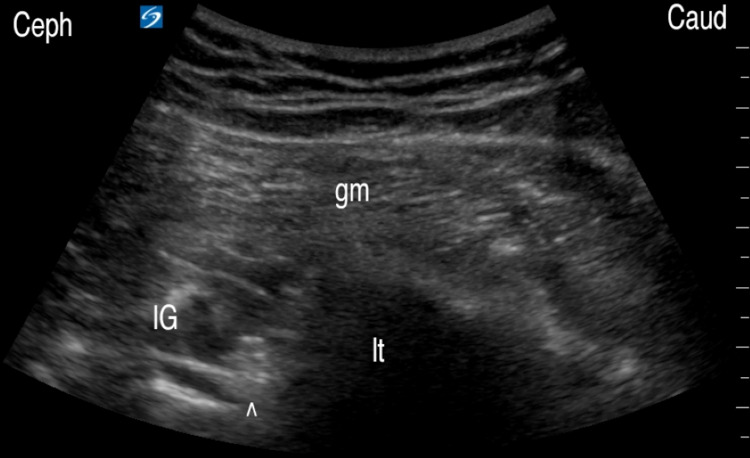
The lesser trochanter is caudal to the GTsn. The lesser trochanter may obstruct the path of the caudal-to-cephalad approach of the needle or RF probe. GTsn: greater trochanteric sensory nerve; RF: radiofrequency; QF: quadratus femoris ^: inferior gemellus (IG), GTsn; ←: lesser trochanter (lt), needle/probe

Several factors need to be optimized for safe and successful RFA probe placement. First, because of the depth of the GTsn, the patient’s body habitus needs to be taken into account in the selection of the RF probes. The probe is inserted more caudally at the gluteal fold to minimize the steep angle of approach and improve ultrasound visualization. Second, the window of approach is bordered by the sciatic nerve medially and the lesser trochanter laterally. This window may be narrow due to individual anatomical variations; furthermore, the lesser trochanter may obstruct the RF probe approach inferiorly when in the medially pointing position (Figure 3). These issues can be alleviated by placing the hip in maximum passive internal rotation to move the lesser trochanter from the medially pointing position to a posteriorly pointing position, to remove the lesser trochanter from obstructing the posterior approach, and to widen the window of approach. Moreover, the transducer is placed as laterally as possible to avoid penetrating the sciatic nerve during the probe approach. The sciatic nerve is otherwise safe from the active ablation zone because it is superficial to the QF. Dynamic passive rotation of the hip during the preparation can help identify the optimal hip rotation. Third, given the proximity between the GTsn and the MFCA, the use of color-flow Doppler is warranted to fully visualize the MFCA and place the RF probe away from the MFCA to avoid vascular injury.

While pending validation, we believe this procedure presents a unique opportunity for holistic treatment. Patients with recalcitrant tendinopathies are often unable to progress through physical therapy due to limitation by pain. Similarly, many orthobiologic treatments can temporarily increase pain, and treatment may take several months to reach therapeutic effect. The coupling of RFA treatment with physical therapy or orthobiologic injection may represent the most ideal combination to yield both immediate pain relief and long-term functional improvement, allowing rapid improvement of both pain and function.

## Conclusions

The case presented here shows that the novel treatment of GTPS by GTsn RFA may be a viable treatment for recalcitrant GTPS. Our technique allows highly accurate and selective denervation of the GTsn through RFA. This finding is pending further validation with larger clinical studies, as well as head-to-head comparison with current standard treatment modalities to better determine its utility.
